# “Unexplored Continents and Great Stretches of Unknown Territory”

**DOI:** 10.3201/eid2306.AC2306

**Published:** 2017-06

**Authors:** Byron Breedlove

**Affiliations:** Centers for Disease Control and Prevention, Atlanta, Georgia, USA

**Keywords:** art science connection, emerging infectious diseases, art and medicine, about the cover, unexplored continents and great stretches of unknown territory, Golgi stain, Ramón y Cajal, Calyx of Held, central nervous system, public health

**Figure:**
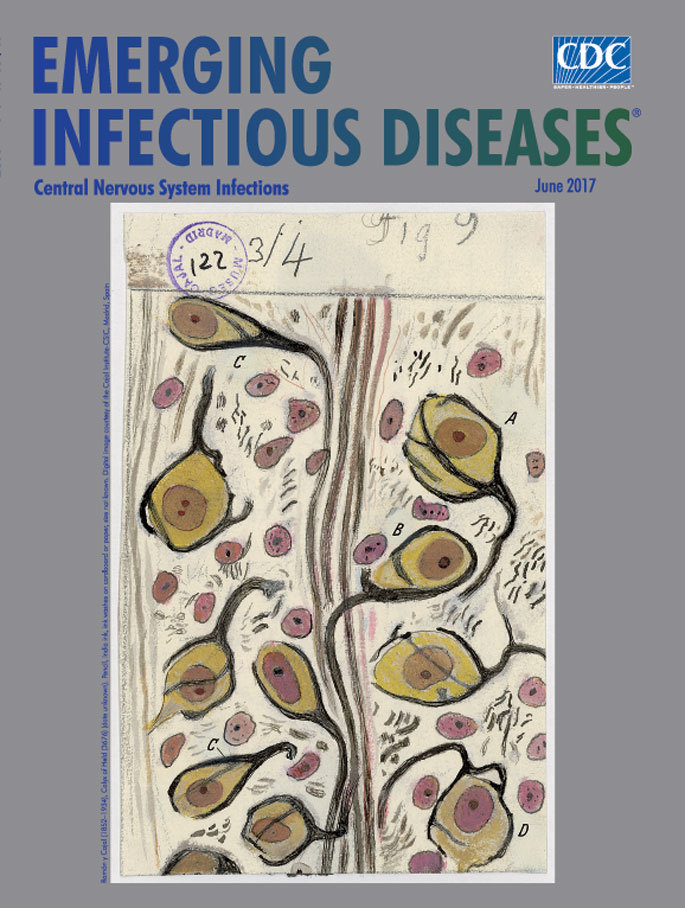
**Ramón y Cajal (1852–1934), Calyx of Held (3676) (date unknown). Pencil, India ink, ink washes on cardboard or paper.** Digital image courtesy of the Cajal Institute-CSIC, Madrid, Spain

This month’s cover art could be, at first glance, an image conjured by the vision of a surrealist painter in the 1920s or an intricate detail lifted from a textile designed by British artist William Morris in the 1870s. It borrows facets from both but is neither. Rather, this striking image is one among the thousands of scientific images created by Santiago Ramón y Cajal, one of the most influential neuroscientists of all time.

Cajal was born in Petilla de Aragón, a small village in northeastern Spain. He is well known for many key contributions to our knowledge of the structure of the nervous system and especially of the brain and spinal cord. Cajal’s career as a researcher, teacher, and mentor spanned more than 50 years, and he published numerous groundbreaking books and more than 100 articles in French and Spanish scientific periodicals. He was also an artist, photographer, bodybuilder, chess player, and publisher.

A eureka moment in Cajal’s career occurred in Madrid in 1887 when another scientist showed him the Golgi stain that made microscopic structures in brain tissue samples appear black against an amber background. Although Golgi’s process was imprecise and inconsistent, Cajal realized that this method could be his pathway to studying microscopic details of the nervous system. After he refined and stabilized the black reaction, he logged long hours at his microscope painstakingly scrutinizing central nervous system tissues from animals and humans. Cajal eventually made more than 3,000 exquisitely detailed sketches of these images, and scientists and educators are still using them.

This month’s EID cover art is Cajal’s drawing of the calyces of Held—so named because Hans Held described the structure’s resemblance to floral calyces in his 1893 article—which is one of the largest synapses in the human brain stem and is integral to the ability to detect and localize high-frequency sounds. Cajal portrayed the calyces as individual cells, encircled by thick black lines that show the axons. From this perspective, the calyces resemble flowers or seedpods intertwined about a central stalk.

Each dot, dash, or line, and the precise patterns, tones, and density in the image are in keeping with Cajal’s credo that “A graphic representation of the object observed guarantees the exactness of the observation itself.” (Note that the handwritten “Manzano number” in the upper left encircled by the stamped words “Museo Cajal Madrid” is a cataloguing convention added by the librarian who inventoried Cajal’s archive in the 1940s.)

In *The Beautiful Brain: The Drawings of Santiago Ramón y Cajal,* Lyndel King and Eric Himmel describe how Cajal developed his sketches: “He preferred to work freehand, rarely resorting to a camera lucida, a device that projects the image from a microscope onto paper where it can be traced. He might start a drawing in pencil, and then later go over it in India ink, adding ink washes or watercolors for tonal areas.”

Cajal’s many distinctions include winning, along with the Italian histologist Camillo Golgi, the Nobel Prize in Physiology or Medicine in 1906. His legacy endures through the continued work and influence of the Spanish school of neurology in Madrid, which developed from his work and vision. King and Himmel offered this assessment of Cajal’s scientific sketches: “When we look at his drawings today, we see not diagrams or arguments, but the first clear pictures of that remote frontier, drawn by the man who traveled farthest into its endless reaches.”

Cajal’s detailed work studying and documenting the complexities of the central nervous system and its myriad pathways, connections, and function remains essential to current research focused on understanding and treating infections of the central nervous system. This system may be either the main target or a secondary target for infections that can result in result in lethal, catastrophic, and debilitating illness.

The catalog of bacteria, viruses, fungi, parasites, and prions responsible for central nervous system disorders spans the alphabet from *Angiostrongylus* to Zika virus. Though we have learned a great deal about how to detect and treat central nervous system infections, the global burden of death and illness underscores the value of public health efforts to prevent, diagnose, and treat them. As Cajal said, “The brain is a world consisting of a number of unexplored continents and great stretches of unknown territory.”
